# Overcoming behavioral variability in electromyography signals by an adaptive incremental classification approach

**DOI:** 10.1016/j.csbj.2025.09.035

**Published:** 2025-10-16

**Authors:** Hiba Hellara, Oumayma Kahouli, Sawsan Njeh, Ahmed Yahia Kallel, Olfa Kanoun

**Affiliations:** Professorship of Measurement and Sensor Technology, Technische Universität Chemnitz, Reichenhainer Str 70, Chemnitz, 09126, Saxony, Germany

**Keywords:** Electromyography, Incremental learning, Adaptive learning, Classification

## Abstract

Surface electromyography (sEMG)-based hand gesture recognition systems often experience significant performance degradation when deployed in heterogeneous populations, particularly among individuals with lifestyle-related physiological variations such as smoking or alcohol consumption. These variations induce distributional shifts that conventional models fail to address, reducing their practical applicability. To overcome this challenge, we introduce an adaptive incremental k-Nearest Neighbors (ADINC-kNN) algorithm designed to maintain robust classification performance without requiring full model retraining. The proposed method integrates a sliding-window buffer with distance-weighted voting to dynamically refine decision boundaries, enabling smooth adaptation from baseline populations (non-smokers/non-drinkers) to target groups (smokers/alcohol consumers). Extensive evaluation was conducted on 15 hand force exercises performed by 14 subjects using 5-fold cross-validation. ADINC-kNN consistently outperformed static kNN, achieving substantial improvements in accuracy, precision, recall, and F1-score across target populations, with classification performance exceeding 90 % in both smoking and alcohol groups. Although the prediction time per fold increased, the algorithm achieved a superior balance between computational efficiency and predictive accuracy compared with state-of-the-art approaches that typically rely on costly retraining. These results demonstrate the effectiveness of ADINC-kNN as a scalable and practical solution for robust sEMG-based gesture recognition. Its ability to adapt dynamically to population-specific physiological variations makes it particularly suitable for real-world applications in rehabilitation, assistive technology, and human–machine interaction, where user diversity and changing conditions are unavoidable.

## Graphical Abstract

**Figure:**
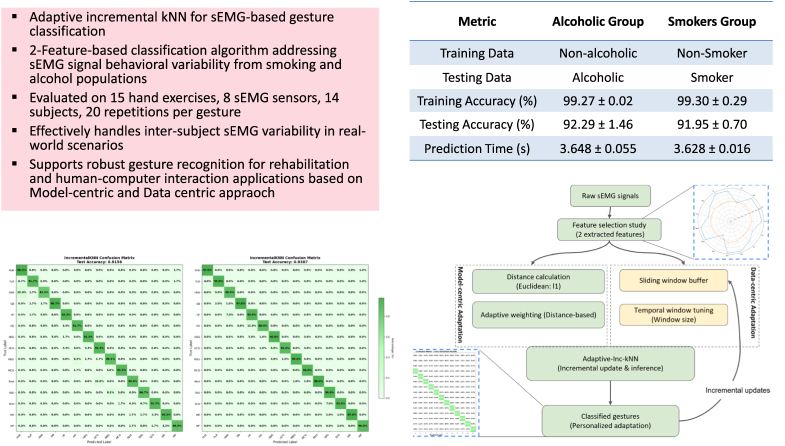

## 1 Introduction

Hand gesture recognition represents a fundamental approach to human-computer interaction that leverages motor control pathways to establish communication between humans and machines. Surface electromyography (sEMG)-based systems capture myoelectric signals generated by motor unit action potentials during voluntary muscle contractions, enabling applications in virtual reality, sign language interpretation, robotic control, and assistive technologies [1], [2]. Unlike vision-based approaches, sEMG systems directly measure neural drive to muscles, providing a physiological representation of motor commands from the central nervous system. The measurement principle is based on different activation patterns of forearm muscle groups during specific hand gestures, producing characteristic myoelectric signatures with amplitudes ranging from μV to millivolts and frequency components between 10–500 Hz. However, achieving robust gesture classification presents significant challenges due to intersubject variability in muscle fiber composition, subcutaneous tissue thickness, skin impedance, and anatomical geometry [3]. In practice, these anatomical and tissue level differences modulate both amplitude and frequency domain features. For example, lower muscle fiber conduction velocity shifts spectral metrics such as median/mean frequency (MDF/MNF) toward lower values. Lifestyle factors, including chronic smoking and alcohol consumption, produce well-documented alterations in neuromuscular function. Chronic smoking impairs muscle physiology through nicotine-induced vasoconstriction, reducing perfusion to skeletal muscles and causing 15–20 % reductions in grip strength compared to controls [4], [5]. Alcohol consumption affects neuromuscular control through central nervous system depression and peripheral muscle impairment, with effects persisting 36–60 hours post-consumption [6]. These physiological changes introduce systematic variations in motor unit firing rates and recruitment patterns that differ from random inter-subject variations in healthy populations. Consistent with these mechanisms, smokers tend to demonstrate lower MDF/MNF and greater spectral fatigue slopes during repeated movements, along with reduced RMS for a given force, reflecting microvascular and excitation contraction constraints. In contrast, recent alcohol exposure manifests more strongly in the temporal domain, as evidenced by longer reaction times, greater onset variability, and elevated RMS/force scaling due to diminished central drive, rather than a consistent spectral shift. Current adaptive approaches exhibit a critical limitation: validation exclusively on healthy populations using standardized datasets such as NinaPro [3], [7], [8], [9], [10]. The signal modifications introduced by lifestyle factors represent consistent patterns of neuromuscular impairment rather than random variations, potentially requiring specialized adaptation mechanisms. We propose an adaptive incremental learning framework for sEMG-based gesture recognition that integrates new data while preserving previously acquired knowledge. This approach maintains classification accuracy across physiologically diverse user groups, including smokers, nonsmokers, alcohol users, and abstainers, by continuously adapting to lifestyle-induced physiological variations, enhancing system robustness and extending applicability to more realistic and inclusive populations. The remainder of this paper is organized as follows: Section 2 reviews related work on gesture classification and incremental learning. Section 3 describes the dataset, subject grouping, and pre-processing techniques. Section 4 outlines the proposed framework and presents experimental results. Section 5 concludes with directions for future research.

## 2 Related research

Surface electromyography has emerged as a cornerstone technology for non-invasive human-computer interaction, with applications spanning prosthetic control, rehabilitation robotics, and assistive technologies [2]. The field has evolved from traditional signal processing approaches to sophisticated machine learning frameworks, each addressing specific limitations while introducing new challenges related to signal variability and cross-user generalization. Early sEMG classification systems relied on handcrafted features extracted from time-domain, frequency-domain, and time-frequency representations [11], [12]. While computationally efficient and interpretable, these approaches suffered from poor generalization due to substantial inter-subject variability in EMG signals, which stems from multiple physiological factors including muscle fiber composition, subcutaneous tissue thickness, and electrode-muscle geometry differences [3], [9]. The classification paradigm divides into user-dependent and user-independent approaches. User-dependent systems construct subject-specific classifiers through individualized training protocols, demonstrating superior performance within their trained domain but exhibiting poor cross-subject transferability due to overfitting [13]. User-independent systems aim to learn generalizable feature representations that remain invariant across subjects. Recent advances in deep learning have revolutionized EMG pattern recognition by enabling automatic feature learning from raw signals. Convolutional neural networks have shown particular promise in learning hierarchical representations that capture both local temporal patterns and broader signal structures [14], [15]. These approaches have achieved classification accuracies exceeding 90 % on standardized benchmarks such as the NinaPro database [16]. However, deep learning models often exhibit poor transferability between subjects due to overfitting to subject-specific signal characteristics [17], [18]. This limitation has motivated adaptive and incremental learning strategies. Transfer learning and domain adaptation techniques have emerged as promising directions, with Du et al. [9] pioneering domain adaptation using adversarial training to learn user-invariant features, achieving 15–20 % improvement in cross-user accuracy.

Recent work by Zheng et al. [3] demonstrated significant advances in adaptive k-nearest neighbors approaches for cross-user EMG adaptation, achieving 68–83 % accuracy across different gesture types using muscle synergy features and risk evaluation mechanisms. Their approach addressed inter-subject variability through representative sample extraction, adaptive weight updating, and intelligent training set management, incorporating a risk evaluator that selectively added high-confidence predictions to enable continuous learning. Incremental learning allows models to acquire new gestures while preserving previously learned knowledge, eliminating the computational burden of complete dataset retraining [19], [20]. Lightweight classifiers such as k-nearest neighbors have been explored for rapid adaptation without retraining [10]. Multimodal approaches combining sEMG with inertial measurement unit data have achieved classification accuracies ranging from 67.5 % to 92.9 % [21].

Despite these advances, current adaptive EMG classification approaches exhibit critical limitations for real-world deployment. Most existing methods focus on general cross-user adaptation without considering specific physiological variations that significantly affect signal quality beyond standard inter-subject differences. Lifestyle factors such as chronic smoking and alcohol consumption impact neuromuscular function through multiple physiological pathways that introduce systematic variations in EMG signal patterns. Chronic smoking affects muscle function through nicotine-induced vasoconstriction, reducing blood flow and limiting oxygen delivery during contractions, manifesting as reduced muscle strength with grip strength reductions of 15–20 % compared to non-smokers [4], [5]. Similarly, alcohol consumption impairs neuromuscular control through effects on both central nervous system and peripheral muscle function, with studies showing significant decreases in voluntary activation and altered EMG characteristics persisting 36–60 hours post-consumption [6].

The existing literature reveals a critical gap in understanding how lifestyle-related physiological variations affect adaptive EMG classification effectiveness. While current approaches have demonstrated success for general cross-user adaptation, no systematic framework exists for handling signal modifications introduced by factors such as smoking or alcohol consumption, representing a significant limitation for deploying inclusive assistive technologies across diverse real-world populations.

This work addresses these limitations by developing a computationally efficient adaptive incremental k-nearest neighbors framework specifically designed to handle lifestyle-related physiological variations in EMG signals. Our approach incorporates specialized mechanisms for accommodating systematic signal changes associated with smoking and alcohol consumption, extending beyond technical methodology to address practical challenges of deploying inclusive assistive technologies that maintain performance across diverse user populations.

## 3 Materials, implementation, and methods

This section outlines the methodology used to classify fifteen distinct hand gestures, each of which is characterized by the varying levels of resistance of the objects used. After data collection and signal processing, the focus shifts to the central goal of this study: the model learning process. This process is structured into two complementary phases. The first phase is data-centric and focuses on input quality and representation. The second phase is model-centric and aims to optimize the adaptive classification approach.

### 3.1 Data collection and visualization

The exercises selected for this study reflect a broad spectrum of natural hand movements to capture meaningful muscle activity patterns. Various resistance-based tools, including therapy balls, circular rings, and flexible objects were used at varying resistance levels (’soft’, ’medium’, and ’hard’) to elicit distinguishable sEMG patterns and enable adaptive scaling of effort, imitating real-world usage scenarios.

**Fig. 1:**
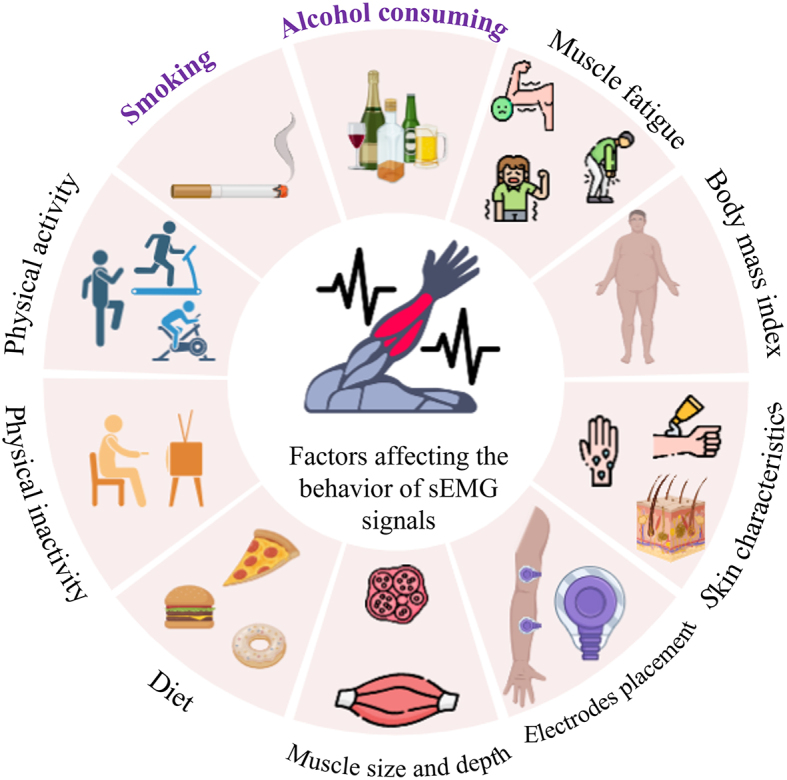
Overview of the factors affecting the behavior of sEMG signals.

The quality of sEMG signals is influenced by subject-specific and methodological factors, as shown in Fig. 1. Physiological characteristics such as muscle fatigue, skin properties, and body composition significantly affect signal amplitude and signal-to-noise ratio [22], [23]. Proper skin preparation, accurate electrode placement on eight superficial forearm muscles [24], and inclusion of rest periods between trials are critical to minimizing artifacts and preventing fatigue-related signal degradation [25], [26].

Fourteen healthy subjects (seven males and seven females) with an average age of 25.14 ± 2.8 years and BMI of 24.81 ± 3.6 kg/m^2^ participated in data collection, as illustrated in Table 1.

**Table 1:** Characteristics of data collection subjects.

Five participants were smokers (35.7 %) and three reported regular alcohol consumption (21.4 %), enabling investigation of lifestyle effects on sEMG patterns. All subjects refrained from consuming food or coffee 2–3 hours before collection [27]. Each participant performed each exercise 20 times using MyoWare sEMG sensors [1] which recorded at 500 Hz, collecting 2000 data points per trial.

**Fig. 2:**
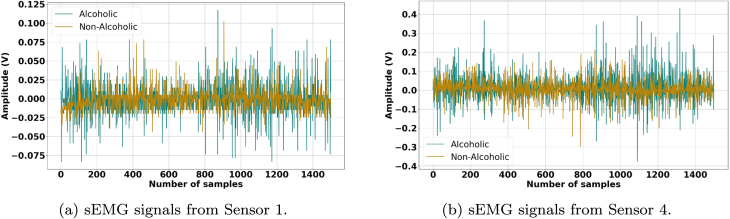
Comparison of sEMG Signals During Hard Ball Grasping in a Pair of Alcoholic and Non-Alcoholic Participants.

**Fig. 3:**
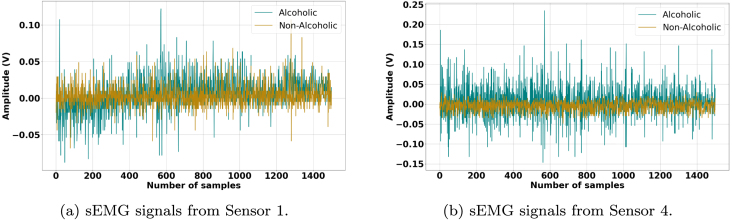
Comparison of sEMG Signals During Hard Ball Grasping in Another Pair of Alcoholic and Non-Alcoholic Participants.

**Fig. 4:**
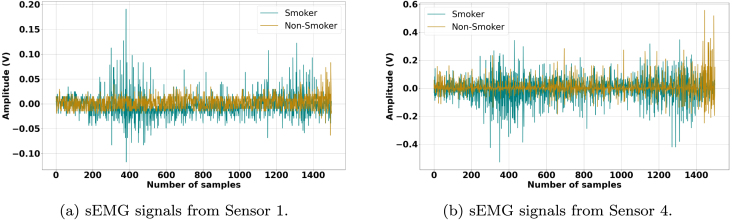
Comparison of sEMG Signals During Hard Ball Grasping in a Pair of Smoker and Non-Smoker Participants.

**Fig. 5:**
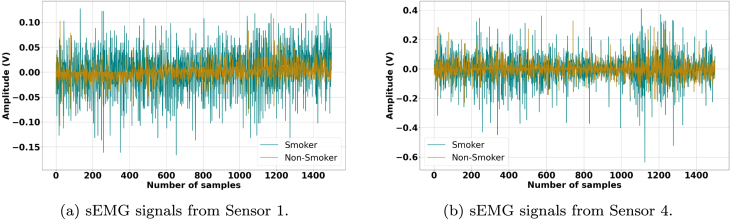
Comparison of sEMG Signals During Hard Ball Grasping in Another Pair of Smoker and Non-Smoker Participants.

Fig. 2, Fig. 3, Fig. 4, Fig. 5 illustrate the influence of alcohol consumption and smoking on neuromuscular activity during hard ball grasping exercises, recorded from Sensor 1 placed on the Brachioradialis muscle and Sensor 4 placed on the Extensor Carpi Radialis Longus muscle. The green traces, representing participants who consume alcohol or smoke, show an earlier onset of fatigue characterized by increased signal amplitude, pronounced fluctuations, elevated baseline noise, and irregular waveform patterns. These signal alterations are consistent with accelerated motor unit recruitment, reduced synchronization efficiency, and unstable firing rates, indicating hallmarks of premature fatigue and impaired neuromuscular coordination.

In contrast, the brown traces from non-consumers and non-smokers display relatively stable and lower-amplitude profiles over the task duration, suggesting more efficient neuromuscular activation, delayed fatigue onset, and sustained motor unit firing regularity. Physiologically, these observations can be attributed to the known effects of alcohol and nicotine on neuromuscular control. Alcohol impairs motor coordination and leads to inefficient muscle activation strategies, requiring compensatory recruitment of additional motor units to maintain grip force. Chronic smoking, on the other hand, compromises peripheral circulation and oxygen delivery, accelerating metabolic fatigue and reducing muscular endurance.

Taken together, these results provide compelling evidence that alcohol consumption and smoking negatively affect muscle control and fatigue resistance during sustained gripping tasks. The observed signal differences emphasize the importance of accounting for such lifestyle factors in sEMG-based assessments of muscle performance and in the development of classification models for biomedical, rehabilitation, and human–machine interface applications.

### 3.2 Data analysis and feature extraction

Given that raw sEMG data are characterized by high levels of noise and inherent complexity, the implementation of robust signal processing and feature extraction techniques is essential to transform these signals into meaningful features for subsequent analysis and classification. The feature extraction methodology employed in this study builds upon our previous research [24], which focused on comparing multiple feature evaluation approaches to identify the optimal feature subset for achieving high performance in classifying fifteen distinct hand force exercises. In the initial phase of the study, a comprehensive set of 37 features was extracted from the sEMG signals. These features were then ranked based on their contribution to classification accuracy. The resulting ranking informed the design of three experimental configurations. The complete set of 37 features is to be considered, in addition to a minimal set comprising only the two highest-ranked features (Wilson Amplitude (WAMP) and Absolute Value of the Summation of Exp Root (AVSER)) and an intermediate set of eight high-ranked features. The following are included: simple square integral (SSI), root mean square (RMS), mean absolute value (MAV), modified mean absolute value 1 (MMAV1), Wilson amplitude (WAMP), root sum of square level (RSSQ), and peak frequency (PKF) [24].

In order to assess the influence of the selected feature set, a simple kNN algorithm with k=3 was employed, using the L1 distance metric. The model parameters were optimized using 5-fold cross-validation. This model was chosen for its simplicity and interpretability, making it a reliable starting point before introducing more sophisticated classifiers or beginning the optimization phase.

As illustrated in Table 2, the two-feature model (WAMP and AVSER) demonstrates superior performance compared to both the full 37-feature set and the 8-feature subset. This superiority is evident in its attainment of a high average testing accuracy of 97.29 % ± 0.20 %, surpassing the 37-feature model (96.52 % ± 0.37 %) and the 8-feature model (96.99 % ± 0.38 %). Moreover, the two-feature model achieves the highest precision (96.63 %), recall (96.60 %), and F1-score (96.56 %), indicating excellent overall classification balance. Importantly, this enhanced performance is accompanied by a substantial reduction in computational cost, with the execution time decreasing to 0.12 s, representing more than a 20-fold speedup relative to the full feature set (2.58 s).

**Table 2:** Comparison of average model performance using different feature sets.

Evaluation metric	37 Features	8 Features	2 Features
**Training Accuracy (%)**	98.41 ± 0.17	98.29 ± 0.18	**96.68**±**0.14**
**Validation Accuracy (%)**	96.08 ± 0.20	96.12 ± 0.19	**96.49**±**0.20**
**Testing Accuracy (%)**	96.52 ± 0.37	96.99 ± 0.38	**97.29**±**0.20**
**Precision (%)**	96.14	96.08	**96.63**
**Recall (%)**	95.99	95.98	**96.60**
**F1-score (%)**	96.00	95.99	**96.56**
**Execution Time (s)**	2.58	0.43	**0.12**

**Fig. 6:**
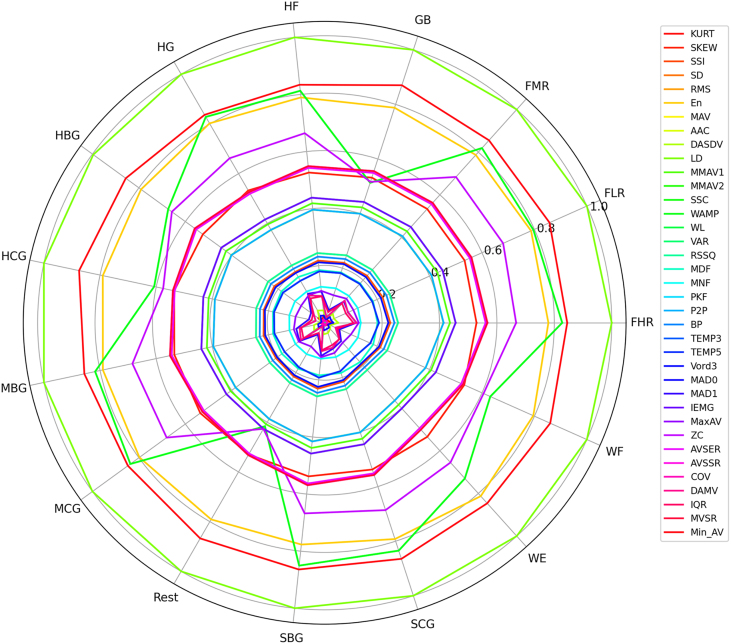
Radar plot 37 features.

**Fig. 7:**
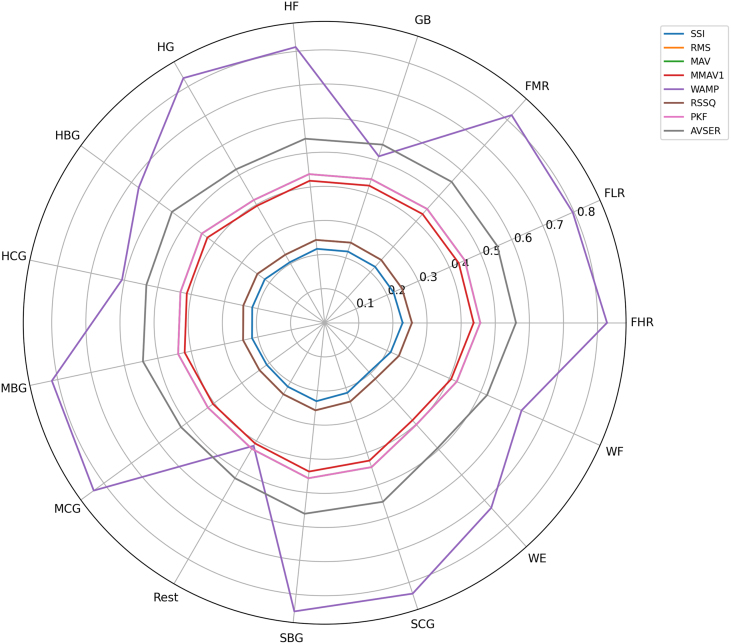
Radar plot 8 features.

These findings underscore the efficacy of strategic feature selection, as WAMP and AVSER alone capture the most discriminative characteristics of the sEMG signals. To further validate the importance of these two features relative to the others, a radar plot visualization across all classes is provided, highlighting their superior discriminative power and consistent contribution to classification performance. The comparison between the radar plots of the full 37-feature set and the reduced 8-feature subset, respectively presented in Fig. 6, Fig. 7, demonstrates that reducing the feature space improves interpretability and enhances the visibility of discriminative patterns across classes. While the 37-feature representation is characterized by redundancy and overlapping behavior, the reduced 8-feature set reveals clearer inter-class differences. Of particular significance is the observation that within this constrained environment, the WAMP and AVSER demonstrate the most substantial variability across the various classes, thereby signifying their remarkable discriminatory capability. This observation underscores the notion that a minimal set of these two well-chosen features can be sufficient to capture the essential differences among classes. Consequently, this provides a robust foundation for classification applications while reducing computational complexity.

Consequently, the resulting model not only sustains exceptional predictive accuracy but also minimizes computational demands, an attribute particularly advantageous for real-time applications and deployment on resource-constrained platforms such as embedded systems and wearable health-monitoring devices.

### 3.3 Traditional hand gesture classification for real-world scenarios

This preliminary evaluation primarily aims to assess the suitability of the dataset for classification tasks and to investigate how physiological characteristics, particularly alcohol consumption and smoking status, influence model performance. To ensure a fair and reliable assessment, an inter-subject evaluation strategy was employed for all experiments, including both the full dataset and the subgroup analyses. Specifically, the dataset was first randomly divided into training (70 %) and testing (30 %) subsets at the participant level, ensuring that data from the same subject did not appear in both sets. Within the training subset, a 5-fold cross-validation procedure was performed to optimize and evaluate model performance across multiple splits, reducing potential bias and improving generalization. This entire process was repeated 10 independent times with random shuffling in each run to account for variability and to obtain robust average results.

**Fig. 8:**
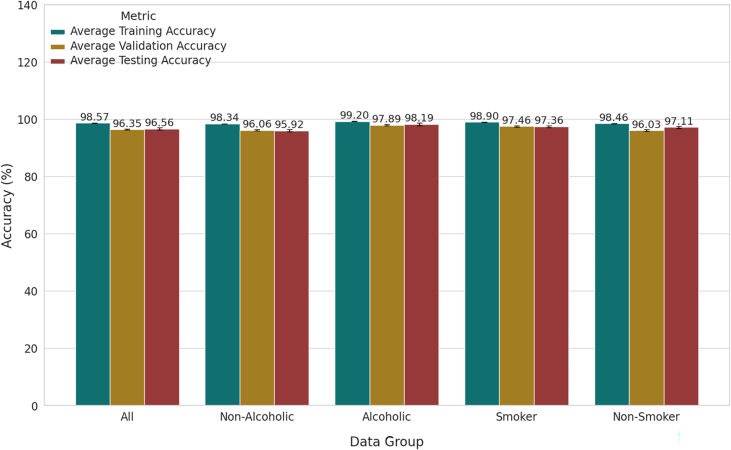
Training, Validation, and Testing Average Accuracies by Dataset Group.

As illustrated in Fig. 8, a series of classification experiments was conducted by segmenting the dataset into five distinct groups based on participants’ profiles: the full dataset (“All”), Non-Alcoholic, Alcoholic, Non-Smoker, and Smoker samples. The bar chart reports the mean training, validation, and testing accuracies obtained across the repeated inter-subject evaluations. This methodology ensures that the comparisons between groups are fair, as all groups are subjected to the same inter-subject testing protocol, allowing a meaningful interpretation of how alcohol consumption and smoking status affect the classification performance.

It is noteworthy that the classifier consistently achieved high training and validation accuracies across all groups, indicating that the model adequately fits the training data without signs of underfitting. However, the testing accuracy, which reflects the model’s ability to generalize to unseen data, exhibited some variability between groups. The “All” group, which utilizes the complete dataset, produced a balanced performance with an average testing accuracy of 96.56 % ± 0.43 %, representing a reasonable baseline for comparison. Interestingly, the Alcoholic group achieved the highest overall performance, with an average testing accuracy of 98.19 % ± 0.48 %, coupled with high training (99.20 % ± 0.03 %) and validation (97.89 % ± 0.26 %) accuracies, suggesting strong generalization despite being trained on a smaller, more homogeneous subset of the data. In contrast, the Non-Alcoholic group yielded the lowest testing accuracy (95.92 % ± 0.37 %), indicating slightly weaker generalization capability compared to the other groups. Similarly, when stratifying the data based on smoking status, the Smoker group achieved higher testing accuracy (97.36 % ± 0.38 %) than the Non-Smoker group (97.11 % ± 0.46 %), despite similar training and validation accuracies. This finding suggests that the sEMG features from smokers may provide slightly more discriminative information, leading to improved classification robustness. Furthermore, these results indicate that population-specific models can outperform models trained on the entire dataset, particularly for the Alcoholic and Smoker groups. The variability observed across groups can likely be attributed to differences in sample size, physiological signal characteristics, and inter-subject variability, underscoring the importance of considering demographic and lifestyle factors in model evaluation.

The preliminary evaluation of the dataset using a basic kNN classifier indicates that the data is generally stable for classification tasks. Nevertheless, a more in-depth analysis reveals important limitations in the model’s ability to generalize across physiologically distinct subpopulations. To examine this issue, we conducted inter-subject subgroup-specific evaluations with strictly separated training and testing data. For the alcohol-consumption scenario, the model was trained and validated on data from 11 non-alcoholic subjects (78.6 % of the dataset) and tested on data from 3 alcoholic subjects (21.4 %). Similarly, for the smoking scenario, training and validation were performed on data from 9 non-smoker subjects (64.3 %), while testing was conducted on data from 5 smoker subjects (35.7 %). This setup ensures that training and testing were carried out on completely distinct participants, providing a rigorous assessment of the model’s capacity to generalize across these physiologically distinct groups.

To ensure robust evaluation and avoid data leakage, we implemented a subject-wise 5-fold cross-validation procedure. In this approach, subjects are first identified and assigned unique IDs based on their recorded samples, guaranteeing that no subject’s data appears in both the training and testing sets. The subjects are then distributed across five folds in a balanced manner to maintain a representative mix of alcoholic and non-alcoholic participants in each fold. For each fold, the model is trained exclusively on data from the remaining four folds and evaluated on the held-out fold, thus performing a strict inter-subject validation.

**Fig. 9:**
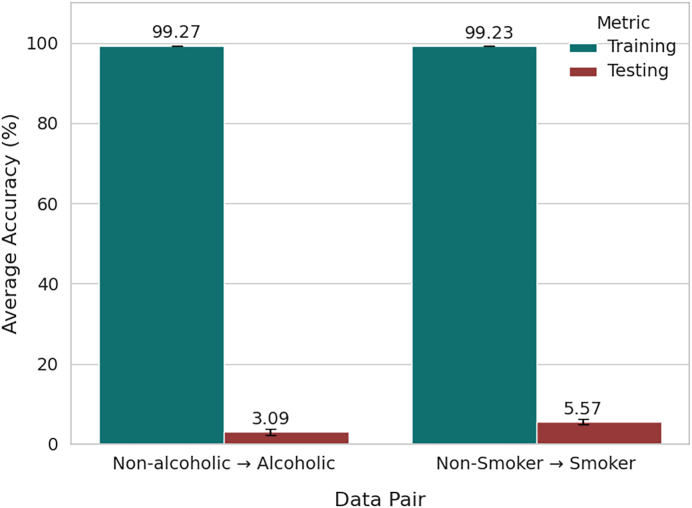
Average Trainin and Testing Accuracies over Train and Testing Data Diversity.

As illustrated in Fig. 9, the traditional kNN model achieved extremely high average training accuracies for both subgroups: 99.27 % ± 0.02 % for alcoholics and 99.23 % ± 0.01 % for smokers. These results indicate that the model was able to effectively memorize the intra-group patterns during training. However, when evaluated on contrasting subgroups, the average testing accuracies dropped drastically to 3.09 % ± 0.80 % for alcoholics and 5.57 % ± 0.65 % for smokers. This stark performance degradation reveals a severe generalization gap, implying that the learned decision boundaries fail to transfer across populations with different behavioral or physiological traits.

In addition to the low testing accuracy, the precision, recall, and F1-scores for both subgroups remained poor (precision: 23–26 %, recall: 1–11 %, F1-score: 3–13 %), further confirming the model’s inability to capture discriminative features across heterogeneous cohorts. The disparity between high training accuracy and weak testing performance underscores a strong overfitting tendency inherent in the conventional kNN approach.

These findings emphasize the critical importance of employing more advanced modeling strategies when analyzing heterogeneous populations. Training models on pooled datasets without accounting for subgroup variability results in overly optimistic performance estimates and limits robustness in real-world scenarios. To address this, subgroup-aware evaluation methodologies and adaptive modeling strategies are essential. In particular, conventional static models such as traditional kNN struggle to adapt to variations between alcohol users and smokers. Consequently, our proposed adaptive incremental kNN method is introduced to continuously update model parameters in response to evolving data distributions, with the goal of improving generalization and robustness across diverse subpopulations.

## 4 Proposed method, results, and discussion

**Fig. 10:**
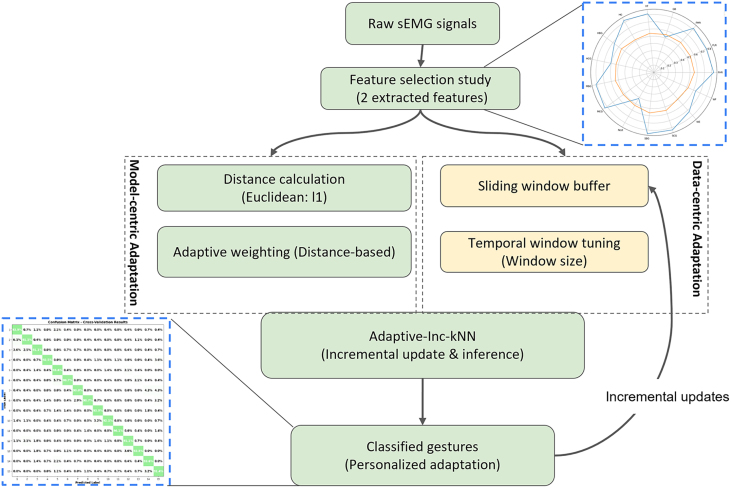
Enhancing sEMG-Based Learning Through Data-Centric and Model-Centric Approaches.

This work introduces an adaptive incremental learning framework for sEMG-based classification that addresses the non-stationarity of biosignals. The novelty of the approach lies in combining data-centric temporal buffering with model-centric distance weighting, enabling the system to adapt continuously to evolving input distributions while remaining computationally efficient. The overall architecture is illustrated in Fig. 10, and the detailed procedure is demonstrated in Algorithm 1.

### 4.1 Learning framework: data-centric and model-centric approach

Algorithm 1 **Adaptive-Incremental-kNN (ADINC-kNN)**

**Fig. 11:**
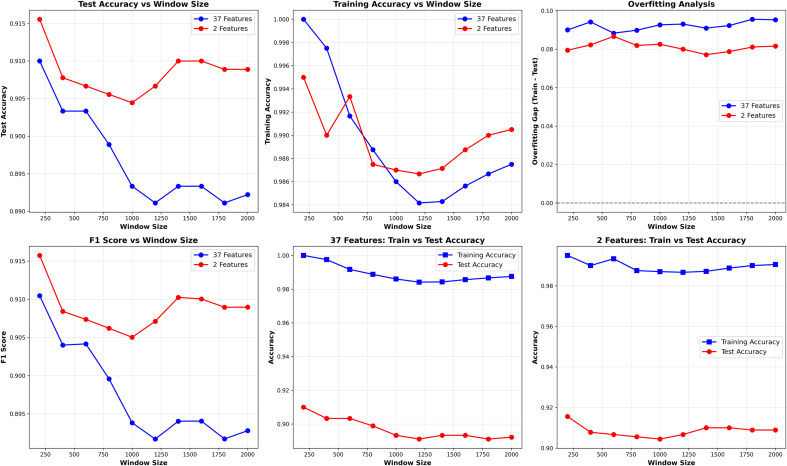
Impact of Sliding Window Size on Learning Performance.
• Model-centric adaptation: During inference, classification is performed using a Distance-Weighted k-Nearest Neighbors (DW-kNN) model with either $\ell_{1}$ or Euclidean distance metrics, respecting the following equation:
  $$w_{i}=\frac{1}{d(x,x_{i})+\varepsilon },$$
  where
  • $w_{i}$ is the weight assigned to the i-th neighbor,
  • $d(x,x_{i})$ is the distance between the query point x and the neighbor $x_{i}$,
  • ε>0 is a small constant added to avoid division by zero.
  This relevance-aware weighting enhances the classifier’s sensitivity to local structures in the feature space, which is particularly important given the dimensionality reduction applied in this study, from 37 features per channel to only 2 key features (Section 3). By giving higher weights to locally relevant patterns, the method reduces the effects of noise and gradual distributional shifts.

The proposed framework, Adaptive-Incremental-kNN (ADINC-kNN), is composed of two complementary adaptation mechanisms:

• Data-Centric Adaptation: To ensure temporal relevance in the highly dynamic sEMG domain, we utilize a sliding window buffer that retains only the most recent training samples. As new labeled instances are added, outdated samples are pruned once the buffer exceeds a predefined size. This incremental update scheme maintains continuity in the training data and supports real-time adaptation to individual subject physiological patterns. Moreover, the window size parameter controls the trade-off between responsiveness which entails short windows, and faster adaptation and stability which is reflected in long windows, and better generalization. The quantitative impact of this parameter is analyzed in Section 4.3.1 (see Fig. 11).

### 4.2 Adaptive-incremental-kNN (ADINC-kNN) algorithm

The ADINC-kNN procedure, summarized in Algorithm 1, begins by initializing an empty buffer that is incrementally updated with newly acquired labeled features. To ensure temporal relevance, the buffer is pruned whenever its size exceeds the window size. A study is conducted to fine-tune the buffer sizes according to the given data in Section 4.3.1. During classification, distances between the query instance and buffered samples are computed, and the final decision is made through distance-weighted voting, where closer neighbors exert stronger influence on the outcome. This design enables the classifier to continuously adapt to evolving biosignal dynamics without requiring full retraining, while remaining lightweight enough for real-time deployment. Collectively, the dual adaptation strategy enhances robustness against temporal fluctuations, inter-subject variability, and distributional drift in sEMG signals.

### 4.3 Results and analysis

This section presents the experimental findings obtained from the proposed approach. The results are analyzed in detail to evaluate model’s performance, robustness, and comparison with existing state-of-the-art methods.

#### 4.3.1 Sliding window-size study

The effect of varying the sliding window size on model performance is illustrated in Fig. 11. Several trends can be observed across evaluation metrics.

First, the test accuracy reveals a trade-off between responsiveness and stability. With smaller windows (<200 samples), the model achieves high accuracy but exhibits instability, particularly when using the full 37-feature set. As the window size increases, accuracy decreases sharply for the high-dimensional case, reflecting the adverse effects of redundant information and noise accumulation. In contrast, with only 2 features per channel, test accuracy stabilizes around 92 % once the window reaches approximately 200 samples, confirming that moderate window sizes preserve temporal relevance without overfitting. Training accuracy further highlights this behavior: small windows produce nearly perfect training accuracy, especially with 37 features, indicating a tendency toward memorization. As the window size grows, training accuracy decreases, aligning with improved generalization but reduced responsiveness. Overfitting analysis underscores these findings. A substantial gap between training and test accuracy is evident for the 37-feature case, particularly with larger window sizes, whereas the reduced 2-feature representation maintains a consistently smaller gap, signaling improved generalization and stability. F1-score follows similar trends to test accuracy. With 2 features, F1-scores peak around a window size of 200–400 samples before plateauing, while with 37 features, the F1-score deteriorates as the window size increases, reflecting the imbalance introduced by high dimensionality. The comparison of train vs. test accuracy in both feature configurations confirms that dimensionality reduction mitigates overfitting, yielding closer alignment between training and test performance across different window sizes. Overall, these results demonstrate that an optimal sliding window size of approximately 200 samples provides the best balance between responsiveness and stability. Furthermore, dimensionality reduction from 37 to 2 features per channel significantly improves robustness, reducing the impact of overfitting and stabilizing performance under streaming conditions. These findings validate the design choice of incorporating temporal parameter tuning into the proposed ADINC-kNN framework.

#### 4.3.2 Evaluation of model accuracy and robustness

To evaluate the robustness of the proposed ADINC-kNN algorithm under real-world domain shifts, we conducted experiments in two behavioral groups: individuals with alcohol use and those who smoke. In each case, the model was trained on data from a baseline population (non-alcoholic or non-smoker) and tested on the corresponding target group (alcoholic or smoker). This experimental setup simulates practical deployment scenarios where models must generalize from healthy individuals to populations with differing physiological characteristics. The average classification performance for these conditions is reported in Table 3.

**Table 3:** Average classification performance of the adinc-knn algorithm with both data groups.

Evaluation metric	Alcoholic group		Smokers group	
**Training Data**	Non-alcoholic		Non-Smoker	
**Testing Data**	Alcoholic		Smoker	
**Model**	**kNN**	**ADINC-kNN**	**kNN**	**ADINC-kNN**
**Training Accuracy (%)**	99.27 ± 0.02	99.32 ± 0.18	99.23 ± 0.01	99.30 ± 0.29
**Testing Accuracy (%)**	3.09 ± 0.80	92.29 ± 1.46	5.57 ± 0.65	91.95 ± 0.70
**Precision (%)**	23.00	95.85 ± 0.99	26.00	95.88 ± 1.49
**Recall (%)**	1.00	92.29 ± 1.46	11.00	91.95 ± 0.70
**F1-score(%)**	3.00	93.97 ± 0.72	13.00	93.81 ± 0.98
**Prediction Time**	0.077 ± 0.028	3.648 ± 0.055	0.069 ± 0.018	3.628 ± 0.016
**per fold (s)**				

Using the optimal temporal buffer determined in Section 4.3.1 (window size = 200), the proposed ADINC-kNN achieved 92.29 ± 1.46 % and 91.95 ± 0.70 % test accuracy on the alcoholic and smoker cohorts, respectively. The corresponding F1-scores were 93.97 ± 0.72 % and 93.81 ± 0.98 %. Precision remained consistently high (approximately 95.9 %), while recall was lower (approximately 92 %), indicating a conservative decision profile where the model tends to minimize false positives at the expense of higher false negatives. Training accuracy reached approximately 99.3 %, which is characteristic of instance-based learners, and reflects an expected ∼7 % generalization gap under cross-group evaluation.

These results were obtained under a 5-fold cross-validation protocol, where the division was performed per subject to ensure subject-independent evaluation and to prevent data leakage between training and testing sets. This validation scheme provides a robust estimate of the model’s ability to generalize across individuals.

**Fig. 12:**
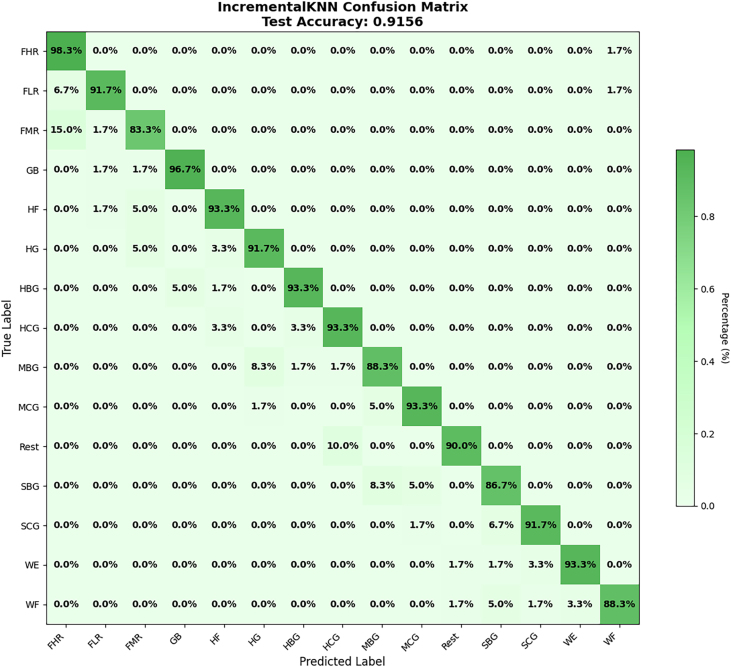
Alcoholic category confusion matrix.

**Fig. 13:**
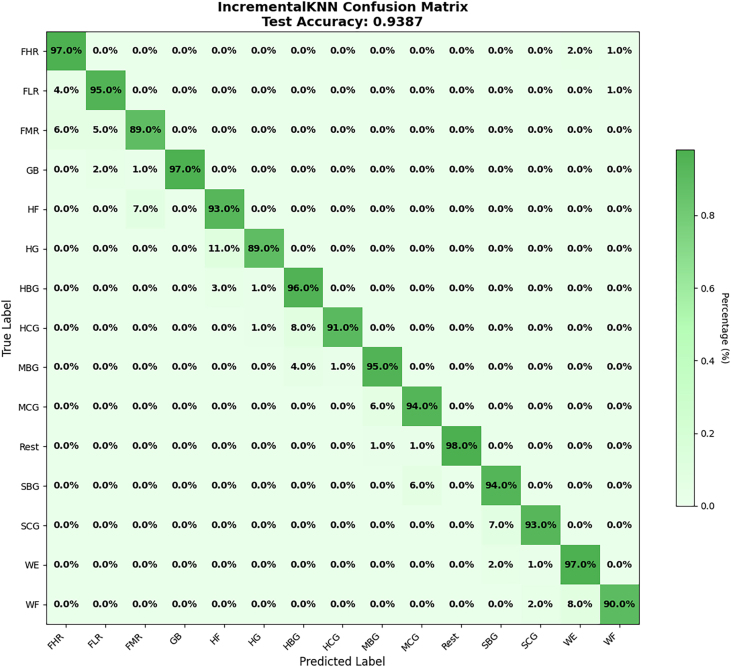
Smoking category confusion matrix.

In addition to the standard metrics (accuracy, precision, recall, and F1-score), we further evaluated performance using the confusion matrix as an error-distribution measure. The confusion matrix highlights how misclassifications were distributed across gesture classes, offering deeper insights into the nature of errors and the relative strengths and weaknesses of the model. Overall, the findings demonstrate that ADINC-kNN can transfer effectively from control-trained models to target cohorts while maintaining real-time feasibility (latency ≈ 3.6 s per fold). The observed precision–recall asymmetry reflects the *model-centric distance-weighted voting*, which emphasizes locally consistent neighbors, while the *data-centric sliding window* ensures the model adapts to contextually relevant recent samples, see Fig. 12, Fig. 13.

#### 4.3.3 Comparison with state-of-the-art approaches

Table 4 compares the proposed ADINC-kNN with recent adaptive EMG classification methods. Our method achieved an accuracy of 92.33 ± 1.08 %, matching the best-performing T-SVM (92.33 ± 1.42 %) and outperforming TI-SVM (87.67 ± 1.63 %) and CNN-based fine-tuning (82.78 ± 2.53 %). Importantly, ADINC-kNN has a lower standard deviation than T-SVM, indicating more stable performance across folds. This stability is a crucial criterion for real-time EMG-based applications, where consistency is as important as average accuracy. In terms of computational efficiency, ADINC-kNN balances accuracy with response time (153.61 ms), outperforming CNN-based methods (336.35 ms) while remaining practical for real-time deployment.

**Table 4:** Comparison with State-of-the-Art Adaptive EMG Classification Methods.

Method	Classifier	Accuracy (%)	Response time (ms)
Li et al. (2021) [7]	I-SVM	32.11 ± 3.21	2.9262 ± 0.2371
Li et al. (2021) [7]	T-SVM	92.33 ± 1.42	6.8304 ± 0.5218
Li et al. (2021) [7]	TI-SVM	87.67 ± 1.63	3.9002 ± 0.5198
Ameri et al. (2019) [28]	CNN + Fine-tuning	82.78 ± 2.53	336.3451 ± 19.1295
**Proposed Method**	**ADINC-kNN**	**92.33**±**1.08**	**153.6141**±**7.5204**

**Fig. 14:**
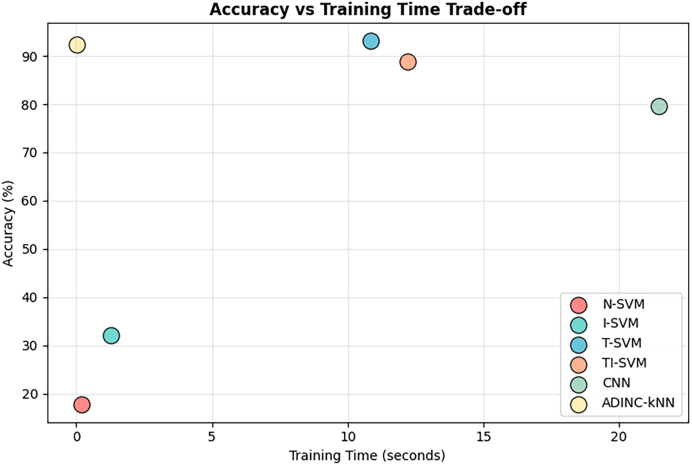
Comparison of training time trade-off.

For more deeper investigation, Fig. 14 illustrates the trade-off between accuracy and training time for different classification algorithms. While traditional SVM variants (N-SVM, I-SVM, T-SVM, TI-SVM) and CNN either suffer from lower accuracy or significantly higher training times, the proposed ADINC-kNN method achieves the highest accuracy (≈92%) with near-zero training time. This demonstrates a clear improvement over state-of-the-art methods, which require substantially longer training durations to achieve comparable or lower accuracy.

### 4.4 Technical contributions and innovations

The primary technical contribution lies in the development of a computationally efficient adaptation mechanism that addresses systematic physiological variations rather than random inter-subject differences. The sliding window buffer approach provides temporal adaptation capabilities that accommodate the evolving signal characteristics associated with lifestyle-related neuromuscular changes. Unlike representative sample extraction methods that require clustering algorithms and feature space analysis, our approach maintains adaptation efficiency through simple temporal ordering and distance-based replacement strategies.

The feature optimization to two highly discriminative parameters (WAMP and AVSER) represents a significant advancement in computational efficiency for EMG classification systems. This reduction from typical multi-dimensional feature spaces (37 features in traditional approaches, muscle synergy matrices in advanced methods) to two parameters enables deployment on embedded systems and wearable devices while maintaining classification performance. The selected features capture both amplitude dynamics (WAMP) and signal variability characteristics (AVSER) that are particularly sensitive to the neuromuscular changes associated with lifestyle factors. The distance-weighted voting mechanism enhances the basic kNN approach by incorporating proximity-based confidence measures that improve classification robustness under the variable signal conditions characteristic of impaired populations. This weighting strategy proves particularly effective for lifestyle-affected users, whose signal patterns may exhibit greater variability compared to those in healthy populations.

**Fig. 15:**
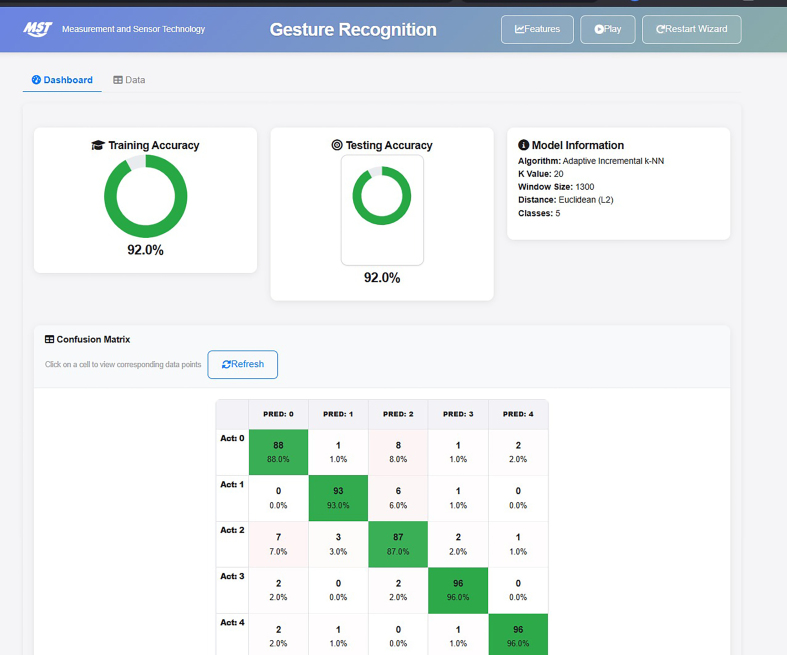
Prototype of the Interactive Gesture Recognition Dashboard.

These algorithmic improvements resulted in a notable boost in classification performance, achieving 92 % training and testing accuracy, as illustrated in Fig. 15. To facilitate adoption by researchers and clinicians, we have developed an interactive dashboard (Fig. 15) that visualizes model performance through accuracy metrics, confusion matrix, and model parameters. The dashboard also allows users to refresh results and test new data without programming expertise, making it a practical step toward a future web-based platform that will support large-scale validation and clinical translation of this approach.

## 5 Conclusion and future directions

This study proposes an adaptive incremental learning algorithm based on k-Nearest Neighbors (ADINC-kNN) to address the challenge of classifying hand gestures independently of lifestyle factors such as alcohol consumption and smoking. These factors introduce variability in surface electromyography (sEMG) signals that degrades classification performance. By incrementally incorporating representative information from test subjects during inference through a sliding-window buffer and distance-weighted voting, the model preserves previously learned knowledge while dynamically refining its decision boundaries. This enables improved generalization capability without requiring retraining from scratch.

The proposed method demonstrated substantial performance enhancement, increasing mean accuracy from 5.6 % to 91.9 % for smokers and from 3.1 % to 92.3 % for alcohol users, with comparable gains in precision, recall, and F1-score (up to 94 %). These results underscore the effectiveness of adaptive incremental learning in addressing generalization gaps caused by lifestyle-related physiological variability. The approach shows particular value in healthcare contexts where data are collected longitudinally from diverse populations, supporting the development of reliable, personalized machine learning systems for inclusive human–computer interaction.

While the current evaluation focuses on force-based exercises rather than standard gesture recognition tasks, this reflects functional assessment requirements relevant to rehabilitation and assistive technology applications. The adaptation mechanism currently addresses binary population differences (smoker vs. non-smoker, alcohol vs. non-alcohol users). Future research will generalize these findings by collecting more data from larger and more diverse populations, allowing continuous adaptation strategies that accommodate varying degrees of physiological impairment and multiple concurrent lifestyle factors. In addition, we will further develop and refine the prepared dashboard tool, improving its visualization and real-time feedback capabilities to better support clinicians, researchers, and end-users. The next step will be to finalize and release this tool as a public, web-based platform, enabling broader adoption of our methodology.

## CRediT authorship contribution statement

**Hiba Hellara:** Writing – review & editing, Writing – original draft, Visualization, Validation, Supervision, Project administration, Methodology, Investigation, Formal analysis, Data curation, Conceptualization. **Oumayma Kahouli:** Writing – review & editing, Writing – original draft, Validation, Methodology, Investigation, Formal analysis, Conceptualization. **Sawsan Njeh:** Writing – review & editing, Writing – original draft, Visualization, Methodology, Investigation, Data curation. **Ahmed Yahia Kallel:** Writing – review & editing, Writing – original draft. **Olfa Kanoun:** Writing – review & editing, Resources, Project administration, Funding acquisition.

## Institutional review board statement

The study was conducted according to the guidelines of the Declaration of Helsinki, and approved by the Institutional Ethics Committee of Technische Universität Chemnitz, (reference: V-331–15-GJSensor-13052019).

## Declaration of competing interest

The authors declare that they have no known competing financial interests or personal relationships that could have appeared to influence the work reported in this paper.
